# User personas for exercise rehabilitation behaviors in older patients with stable chronic obstructive pulmonary disease: a qualitative study

**DOI:** 10.3389/fpubh.2026.1847534

**Published:** 2026-06-19

**Authors:** Han Su, Luxin Wang, Ying Chen, Xiaoyue Song, Xiao Ruan, Zihan Wang, Binglin Du, Renhui Lu, Yanfei Liu, Weihong Zhang

**Affiliations:** 1 School of Nursing and Health, Zhengzhou University, Zhengzhou, Henan, China; 2 The Fifth Clinical Medical College of Zhengzhou University, Zhengzhou, Henan, China

**Keywords:** chronic obstructive pulmonary disease, exercise rehabilitation, needs, preferences, qualitative study, social support, user persona

## Abstract

**Background:**

Effective exercise rehabilitation is critical for managing older stable chronic obstructive pulmonary disease (COPD) patients, but the heterogeneity in their exercise rehabilitation behaviors is largely unexplored. This study aimed to characterize distinct patterns of exercise rehabilitation behavior and create user personas to guide tailored interventions.

**Methods:**

Using purposive sampling, 18 patients with stable COPD were recruited from a community health center in Zhengzhou, China. Semi-structured interviews were conducted between July and September 2025. Data were analyzed using conventional content analysis. Persona labels were manually extracted and synthesized to construct user personas, which were then visualized using word clouds and descriptive tables.

**Results:**

The persona label system for exercise rehabilitation behaviors was categorized into six dimensions: perceptions of exercise rehabilitation, current exercise status, social support, psychological status, exercise rehabilitation needs, and accessibility of healthcare resources. Based on this system, four distinct types of user personas were constructed: “Proactive Self-Managed Exercisers”, “Routine Independent Exercisers”, “Routine Independent Exercisers”, and “Fear-Avoidant Resource-Limited Exercisers”.

**Conclusions:**

This study reveals significant heterogeneity in exercise rehabilitation behaviors among older patients with stable COPD. The constructed patient personas enable healthcare professionals to identify the unique needs of each individual and implement precisely tailored strategies to achieve sustained exercise rehabilitation.

## 1 Introduction

Chronic Obstructive Pulmonary Disease (COPD) is a common, preventable lung condition marked by ongoing airflow limitation and symptoms like shortness of breath, cough, and mucus production (1). It has emerged as a major global health challenge and ranks as the fourth leading cause of mortality worldwide, with its burden disproportionately affecting older adults (2). The prevalence of COPD rises sharply with age. Data from China show rates of 21.2% among adults aged 60–69 and 35.5% in those aged 70 and above—a trend that continues to climb with population aging (3). COPD is typically marked by prolonged stable phases interspersed with acute exacerbations. Effective long-term management during stable periods is therefore crucial, especially for older patients who are more vulnerable to functional decline and complications (1). Pulmonary rehabilitation represents a key non-pharmacological treatment for patients with stable COPD. Exercise rehabilitation forms the cornerstone of pulmonary rehabilitation (4), and is critically important for older patients in the stable phase of COPD.

Exercise rehabilitation is a comprehensive process involving the assessment of a patient's physical activity status, the development of an exercise prescription, the provision of training guidance, and ongoing monitoring. Evidence indicates that sustained exercise rehabilitation training can enhance mitochondrial oxygen delivery capacity and oxidative function, alleviating dyspnoea symptoms and improving pulmonary function in patients (5). Additionally, exercise rehabilitation has been proven to increase flow-mediated vasodilation, thereby enhancing patients' cardiovascular function (6). It also contributes to improved immune response by selectively inhibiting pro-inflammatory pathways and promoting anti-inflammatory mechanisms (7). In the long term, these benefits are particularly vital for older adults, as they can help counteract age-related functional loss, reduce the risk of hospital readmission, improve patient quality of life, and promote healthy aging (8). Furthermore, authoritative guidelines recommend that pulmonary rehabilitation can be initiated within 1 month after discharge or 3 weeks after hospital admission, establishing a critical window for exercise rehabilitation intervention (9, 10).

Despite the benefits of exercise rehabilitation being well known, patient adherence remains a significant challenge in both clinical practice and research. For instance, a UK study involving 787 older patients revealed that only 57.1% completed an exercise rehabilitation program centered on aerobic and resistance training (11). While most current research focuses on identifying factors influencing exercise rehabilitation behaviors or adherence in older COPD patients, and while some qualitative studies have deeply analyzed facilitators and barriers, their findings often remain as isolated themes that have not been synthesized into representative patient profiles (12). Moreover, exercise rehabilitation behaviors differ significantly among individuals due to personal factors such as physical condition, exercise capacity, symptom burden, psychological status, beliefs about rehabilitation, family support, and living environment in older patients (13, 14). Consequently, many research and clinical intervention programmes continue to employ standardized, one-size-fits-all exercise prescriptions, overlooking this complex heterogeneity of older patients (15). This standardized approach encounters challenges in addressing the diverse needs of the older adult population, resulting in issues such as low participation rates, poor adherence, and suboptimal rehabilitation outcomes, which consequently restrict the full potential of exercise rehabilitation.

To address the previously mentioned challenges and guided by the philosophy of precision nursing (16), a tool is urgently needed to systematically understand patient differences and create personalized intervention plans. The persona approach is precisely such an innovative, user-centered research methodology, originating from the field of design (17), it integrates multi-dimensional user information (e.g., cognition, psychology, behavior, social support, needs), extracts characteristic labels, further synthesizes label dimensions, accurately identifies different patient types and characteristics, clusters users with similar traits into the same category to generate clearly defined visual personas (18). In the domain of health management, personas have gradually been applied to scenarios such as chronic disease self-management, becoming an effective tool for achieving precision nursing. For instance, Yang et al. (19) conducted a study on patients undergoing home-based cardiac rehabilitation for coronary heart disease, creating five self-management personas, such as “active health management seekers” and “limited knowledge and poor management,” to inform stratified intervention strategies. Similarly, Qiu et al. (20) focused on older adults with chronic multimorbidity in the community, constructing five user personas from the perspective of social participation. Galliford et al. (21) based on a data-driven approach, developed and validated eight personas for type 2 diabetes self-management, thereby facilitating the development of classified intervention strategies for diabetes. This persona approach enables healthcare professionals to intuitively understand patient group traits, needs, and behaviors, thereby transcending standardized interventions and providing a pathway for achieving personalized rehabilitation support.

In summary, there is a significant gap in research that systematically outlines and visually depicts the heterogeneity in exercise rehabilitation behaviors among older patients with stable COPD. Social ecological theory emphasizes that individual behavior is shaped by the interplay of multi-level systems, which are typically categorized into three tiers: the microsystem, mesosystem, and macrosystem (22). The microsystem refers to the individual system, encompassing biological, psychological, and social subsystems that influence individual behavior; the mesosystem comprises small-scale groups, such as families and other social networks; and the macrosystem is primarily composed of larger structures, including organizations, institutions, communities, and socio-cultural contexts. Therefore, this study aims to explore the heterogeneity of exercise rehabilitation behaviors among older patients with stable COPD within the Chinese cultural context, to understand their behavioral characteristics and needs at the micro, meso, and macro levels, and to construct user personas, thereby providing a scientific basis for healthcare professionals to develop stratified, precise intervention strategies. Specifically, this study seeks to answer the following research questions: (1) What are the heterogeneous characteristics of exercise rehabilitation behaviors among older patients with stable COPD in the Chinese cultural context, and how are these characteristics manifested at the micro, meso, and macro levels of the socio-ecological model? (2) What types of user personas can be constructed based on these behavioral characteristics and needs?

## 2 Methods

### 2.1 Study design

This study employed a descriptive qualitative approach to conduct semi-structured interviews with patients. Grounded in the principles of naturalistic inquiry (23), the study aimed to describe the heterogeneous exercise rehabilitation behaviors, support contexts, and rehabilitation needs of older patients with stable COPD in real-life settings. Qualitative description was considered appropriate because the study focused on characterizing practical behavioral variations and developing clinically meaningful behavioral profiles, rather than generating formal theory or interpreting latent meanings. Although the study primarily adopted a descriptive orientation, persona construction also involved applied interpretive elements. Conventional content analysis was used to identify persona label dimensions, followed by cross-dimensional comparison and clustering to construct representative user personas. The consolidated criteria for reporting qualitative research (COREQ in Supplementary File 1) (24) guided this study to ensure scientific rigor.

### 2.2 Study setting and recruitment

This study was conducted at a community health center in Zhengzhou, Henan Province, which functions as a primary public health institution. A purposive sampling method, coupled with the principle of maximum variation, was employed to ensure diversity among participants, such as gender, age, and educational level. Nurses at the community center introduced the study to COPD patients attending their annual health check-ups. Two researchers (SH, WLX) screened interested individuals. The first author (SH) then reviewed those who passed this initial screen against the inclusion and exclusion criteria. Eligible individuals were formally invited to participate in the study.

### 2.3 Inclusion and exclusion criteria

The inclusion criteria were as follows: meeting the diagnostic criteria for COPD as defined by the “2024 Global Strategy for Diagnosis, Treatment, Management, and Prevention of GOLD Chronic Obstructive Pulmonary Disease” (25), and being in a stable phase of the disease, defined as no acute exacerbation within the past month and stable clinical symptoms, age ≥60 years old, and provided informed consent and voluntarily agreed to participate in the study. The exclusion criteria were: individuals with cognitive, memory, or speech impairments that would prevent cooperation, and the presence of severe comorbidities that significantly limit physical activity.

### 2.4 Interview guideline

Data were collected through face-to-face, semi-structured interviews from July 5 to September 30, 2025. The initial interview guide was developed based on social ecological theory, combined with a comprehensive literature review and research team discussions. It was subsequently refined through pilot testing with two patients to identify and address potential issues. The final interview guide (Supplementary File 2) included the following core questions: (1) What is your understanding of exercise rehabilitation? (2) What experiences have you had with exercise rehabilitation? (3) What factors have helped or hindered your engagement in exercise rehabilitation? (4) What are your needs and preferences regarding exercise rehabilitation? (5) What perspectives or suggestions do you have on exercise rehabilitation for older COPD patients?

### 2.5 Data collection

The interviews were conducted by two female nursing postgraduate students (SH, WLX). Both interviewers had received training in qualitative research methodologies and possessed practical clinical experience in caring for COPD patients during their internships. Their enthusiastic and outgoing nature, coupled with strong interpersonal skills, facilitated effective communication during patient interviews. Prior to the formal interviews, the interviewers (SH, WLX) familiarized themselves with the interview guide, practiced questioning techniques, and learned to operate the audio recording equipment. Appointments were scheduled with participants in advance to agree on a suitable time and location. Before each interview, the interviewers introduced themselves and explained the purpose of the study and the audio recording procedure to the participants. Written informed consent was obtained from all participants. Sociodemographic information, including age, gender, marital status, educational level, place of residence, and disease duration, was also collected. Interviews were conducted in quiet, private settings, such as offices within community health centers, with each session lasting between 20 and 40 min. No non-participants presented during the interview. During the interviews, the researchers employed techniques such as summarization, repetition, reflective questioning, and follow-up prompts, while adjusting the content as appropriate. Non-verbal cues and interactional dynamics were documented in field notes. Sample size was guided by the data saturation principle. Data collection and qualitative analysis were conducted simultaneously, allowing the researchers to evaluate saturation using the constant comparative method. New data from each interview were immediately coded and compared within the dimensions of the theoretical framework. After conducting at least 10 sessions, saturation was achieved when three consecutive interviews produced no new codes or information in any individual dimension (26), after which data collection was terminated, resulting in a total of 18 interviews for analysis. No repeat interviews were conducted in this study.

### 2.6 Data analysis

#### 2.6.1 Extraction of persona label dimensions

All audio recordings were transcribed verbatim within 24 h of each interview. The transcripts were returned to the participants for verification to ensure accuracy before being imported into NVivo 11.0 for management and analysis. Data were analyzed using content analysis (27), following the specific steps below (28). (1) Two researchers (SH, RX) repeatedly listened to the recordings and read the transcripts to gain a general understanding. (2) Line-by-line open coding was performed to extract meaning units and generate initial codes. (3) Through continuous comparison and research team discussions, codes with conceptual similarities were synthesized into factual labels. (4) Guided by the Social Ecological Theory, these labels were grouped into higher-order, coherent dimensions, establishing the structural framework for subsequent persona construction. During this qualitative abstraction phase, labels were not assigned numerical weights or statistical priorities; their inclusion was strictly determined by their conceptual relevance and contextual salience to the participants' lived experiences.

#### 2.6.2 Clustering and construction of user personas

Patient personas emerged through the systematic comparison, configuration, and aggregation of cross-dimensional features. The operational pathway consisted of three steps: (1) Individual feature extraction: Based on the established social ecological dimensions, the researchers conducted a case-by-case analysis of all participants. By documenting each individual's specific manifestations across all dimensions, individualized multidimensional personas were constructed. (2) These initial individual profiles were returned to the respective participants for verification and calibration, ensuring the qualitative rigor and authenticity of the descriptive data. (3) Cross-dimensional feature comparison and similarity classification: Through collective research team debriefings, individual personas were systematically clustered via three iterative sub-steps: ① Within-dimension trait categorization: Features under each dimension across all cases were horizontally compared to identify variations. ② Cross-dimensional pattern recognition: The team compared recurring feature combinations across dimensions to identify relatively consistent behavioral patterns among participants. ③ Persona clustering and naming: Individuals sharing highly consistent cross-dimensional configurations were aggregated into distinct user personas, and given descriptive names based on their core features.

Crucially, the final patient personas were strictly mutually exclusive. Clear boundaries were determined by explicit assignment criteria: a participant was definitively assigned to a category only if their core attributes, such as dominant exercise rehabilitation behaviors, psychological status, and social support barriers, matched the defining configuration of that specific subgroup.

#### 2.6.3 Validation and representation of user personas

Any disagreements during the persona assignment phase were resolved through iterative team discussions, and three external qualitative and clinical experts audited the final classifications until achieving 100% consensus.

To visually represent the prominent features of each persona, representative keywords were extracted from their feature configurations. During this visualization phase, keywords were assigned weights based on their frequency and contextual significance within each specific subgroup's sub-dataset, and word clouds were generated using the WordArt tool. Finally, all user personas, along with their word clouds and descriptive labels, were integrated into a comprehensive summary table to facilitate systematic comparison.

### 2.7 Ethical considerations

This study received approval from the Zhengzhou University Life Sciences Ethics Committee on January 17, 2025 (Issue: ZZUIRB2025-10). We conducted the research in accordance with the ethics guidelines set out in the Declaration of Helsinki.

### 2.8 Rigor and reflexivity

The rigor of this study was ensured by adhering to the trustworthiness framework of Guba and Lincoln (29, 30), which encompasses credibility, transferability, dependability, and confirmability. Specifically, to further enhance credibility, a triangulation approach was applied, which included researcher analysis, participant validation, and expert evaluation. Transferability was supported by the provision of a detailed account of the study context, participant selection criteria, participant characteristics, and data collection and analysis procedures. Dependability was achieved through independent cross-coding of transcripts by two researchers. Finally, confirmability was ensured through regular peer debriefing sessions where the research team critically examined the evolving findings to ensure they were rooted in the data rather than researcher biases. Furthermore, reflexivity (31) was maintained by documenting researchers' potential influences, including personal beliefs, professional backgrounds, and preconceptions about exercise rehabilitation prior to data collection. Throughout the research process, reflective field notes and memos were utilized to reduce subjective influences on data interpretation.

## 3 Results

### 3.1 Participant characteristics

A total of 18 eligible older patients with stable COPD participated in the study, including 11 males and 7 females. Participants ranged in age from 60 to 75 years, with a mean age of 66.11 years. Disease duration varied from 1 to 12 years, with an average of 5.56 years. Regarding educational level, 4 participants had a primary school education, 5 had a junior high school education, 4 had a senior high school education, and 5 had a college-level education. In terms of marital status, all participants were married except for one, who was divorced, and one was widowed. In terms of residential distribution, 27.78% of participants were long-term rural residents. Detailed participant characteristics are presented in Table 1.

**Table 1:** Participants' characteristics (*n* = 18).

Characteristics	Mean (SD) or *n* (%)
	Mean (SD)
Age (years)	66.11 (4.85)
Disease duration (years)	5.56 (2.91)
	*n* (%)
Gender	
Male	11 (61.11%)
Female	7 (38.89%)
Educational level	
Primary school	4 (22.22%)
Middle school	5 (27.78%)
High school	4 (22.22%)
College	5 (27.78%)
Marriage status	
Married	16 (88.88%)
Divorced	1 (5.56%)
Widowed	1 (5.56%)
Occupation	
Retired	10 (55.54%)
Self-employed	1 (5.56%)
Farmer	5 (27.78%)
Business manager	1 (5.56%)
Actor	1 (5.56%)
Long-term residence	
Urban	13 (72.22%)
Rural	5 (27.78%)
GOLD grades	
GOLD 1	8 (44.44%)
GOLD 2	10 (55.56%)

*n*, number of participants; SD, standard deviation.

### 3.2 Persona label system for exercise rehabilitation behaviors in COPD patients

**Figure 1:**
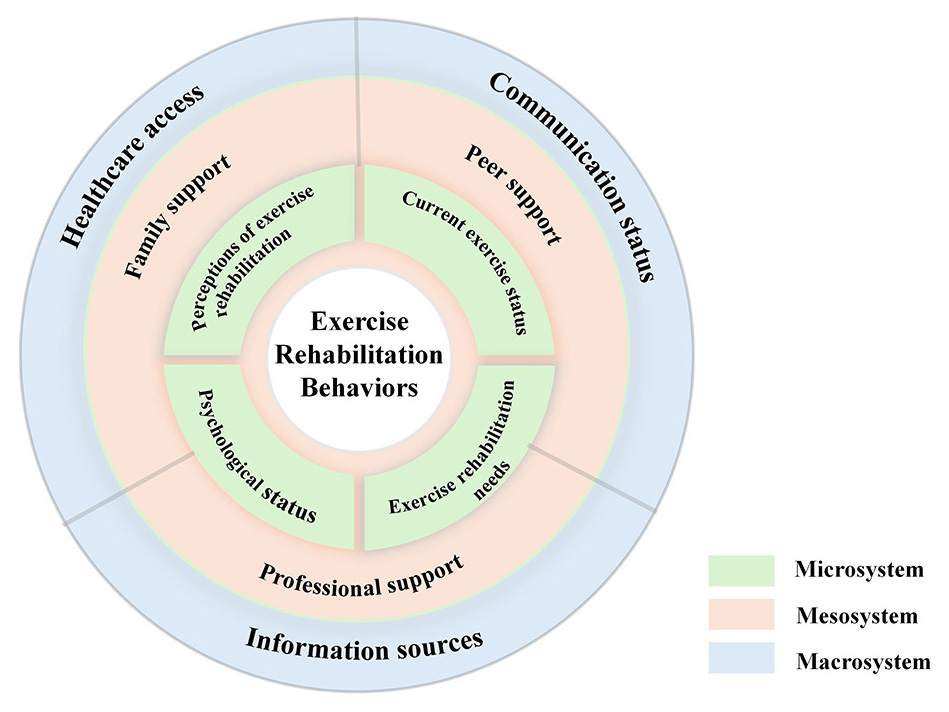
Persona label system mapped to the social ecological model.

Based on the interview findings, a total of 19 labels and 73 codes were identified. These were systematically categorized into six dimensions to form the persona label system for exercise rehabilitation behaviors in COPD patients: perceptions of exercise rehabilitation, current exercise status, psychological status, exercise rehabilitation needs, social support, and accessibility of healthcare resources. The detailed exemplary coding process is provided in Supplementary File 4. These six dimensions correspond to the micro, meso, and macrosystems of the social ecological theory, as illustrated in Figure 1. Notably, participant demographic characteristics (such as age, disease duration, and education level) were not utilized as active clustering variables to construct the personas.

### 3.3 Personas for exercise rehabilitation behaviors in COPD patients

Using the established persona label system, four typical personas for exercise rehabilitation behaviors in COPD patients were developed. Each persona type was visually represented through word clouds generated in WordArt, accompanied by descriptions of dimensional characteristics and integrated with the general sociodemographic profiles of the study participants, as detailed in Table 2.

**Table 2:** (Continued)

Name		Proactive self-managed exercisers	Routine independent exercisers	Family-burdened exercisers	Fear-avoidant resource-limited exercisers
Personas		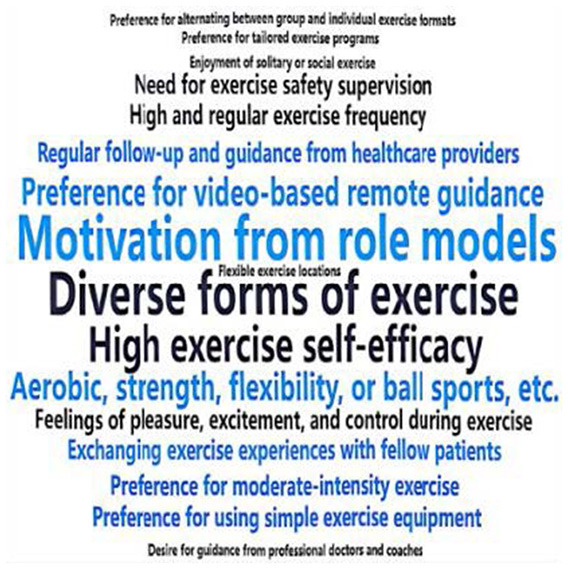	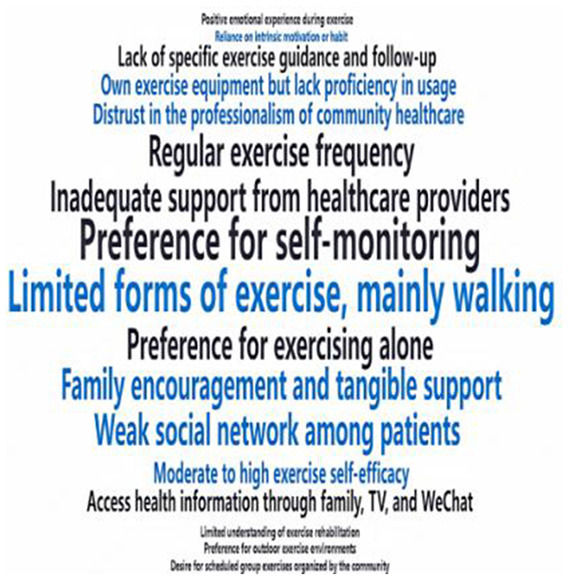	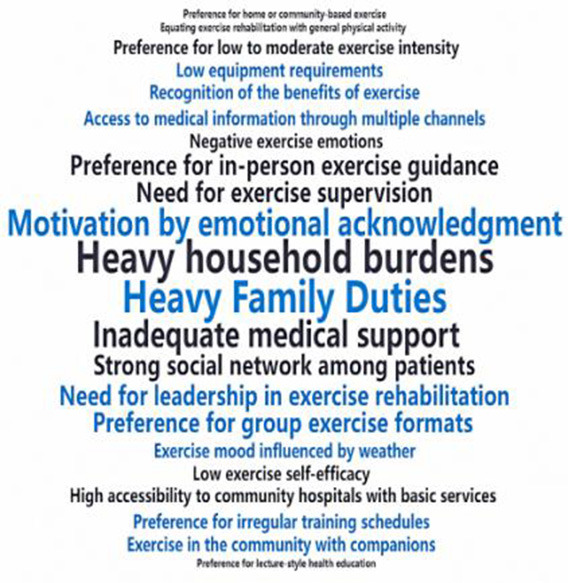	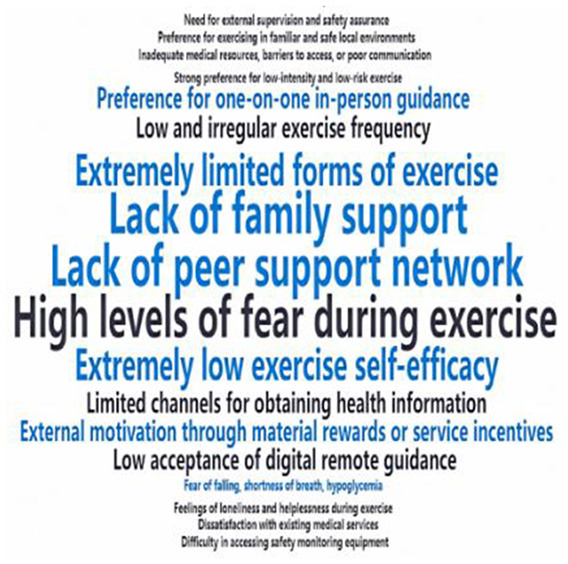
Individuals		P2, P4, P9, P15, P18	P3, P5, P10, P14	P1, P6, P8, P12, P16	P7, P11, P13, P17
Age (years)		61–68	68–75	60–70	60–75
Gender		Male: 3; female: 2	Male: 3; female: 1	Male: 2; female: 3	Male: 3; female: 1
Education level		College: 3; high school: 1; middle school: 1	College: 1; high school: 1; middle school: 2	High school: 2; middle school: 1; primary school: 2	College:1;middleschool:1;primary school: 2
Long-term residence		All urban	All urban	Urban: 3; rural: 2	Urban: 1; rural: 3
Marriage status		All married	All married	All married	Married: 2; divorced: 1; widowed: 1
Disease duration (years)		3–7	8–12	5–8	1–3
GOLD grades		GOLD 1: 3; GOLD 2: 2	GOLD 1: 2; GOLD 2: 2	GOLD 1: 2; GOLD 2: 3	GOLD 1: 1; GOLD 2: 3
Perceptions of exercise rehabilitation	Cognitive depth	Systematic understanding of rehabilitation	Rehabilitation equated with walking	Rehabilitation as daily activities	Vague, unscientific perceptions
	Perceived benefits	Multi-dimensional benefits articulated	General benefits acknowledged	Basic symptom relief recognized	Loss-framed, avoidance-oriented
Current exercise status	Exercise type	Diverse; walking, ball sports, swimming, Baduanjin	Walking predominates; occasional stretching	Limited; mainly walking, household chores	Walking predominates
	Frequency	Highly regular, daily	Regular, daily	Irregular	Highly irregular
	Duration	Moderate duration	Consistent duration	Variable; dictated by daily tasks	Unplanned; no fixed duration
	Locations	Flexible: home, park, gym	Home nearby areas	Home nearby areas	Safe nearby areas
	Companionship	Often with family/friends/peers	Exercises alone	Mostly with neighbors/friends	Exercises alone
Psychological status	Exercise emotions	Positive (pleasure, excitement)	Stable	Symptom-contingent	Fear-dominated, safety concerns
	Exercise self-efficacy	High	Moderate	Low-to-moderate	Low
	Loneliness	None	None, prefers solitary exercise	Avoids loneliness	Profound loneliness
Exercise rehabilitation needs	Modality	Moderate intensity, varied, challenging activities	Low-moderate intensity, simple, executable activities	Low-intensity, daily-integrated activities	Low-intensity, low-balance activities
	Guidance	Professional instruction, video-based remote guidance	Self-monitoring skills, video-based remote guidance	Professional supervision with safety monitoring	Supervision required; prefers in-person over digital
	Motivation	Role models	Internal drive/habits	Family recognition	Material rewards
Social support	Family support	Strong instrumental and emotional support	Good encouragement and practical companionship	Verbal support only; lacks practical companionship	Absent (widowhood/divorce/children afar)
	Peer support	Active peer engagement	Minimal peer exchange	Unstable peer support	Unable to connect with peers
	Professional support	Comprehensive	Limited	Largely absent	Absent
Accessibility of healthcare resources	Healthcare access	Comprehensive; community + tertiary dual support	Basic community care, low trust in community	Basic community care	Significant access barriers
	Communication status	Smooth interaction; multiple contact channels	Poor communication channels with professionals	Poor communication channels with professionals	Blocked communication channels
	Information sources	Healthcare providers, peers, short videos, TV	Family, TV, WeChat	TV, peer communication	Isolated, passive, one-way

#### 3.3.1 Proactive self-managed exercisers

This subgroup primarily consists of urban residents with relatively higher education levels and disease durations of 3–7 years. Their most distinguishing characteristic is a high level of scientific knowledge regarding exercise rehabilitation and proactive, autonomous exercise behaviors. These patients appreciate exercise rehabilitation principles, understand its respiratory benefits, and can identify aerobic, resistance, and flexibility training types. In terms of current exercise practices, their activities are diverse, encompassing walking, ball sports, swimming, and traditional mind-body exercises such as Baduanjin. Exercise frequency is highly regular, with moderate duration and flexible locations. They often exercise with family, friends, or fellow patients, having strong exercise habits and positive social interactions. Furthermore, this group benefits from a robust social support system. Family members provide both emotional encouragement and behavioral companionship, peer networks enable exercise experience sharing, and healthcare professionals offer accessible follow-ups and consultations. Psychologically, they exhibit positive attitudes toward exercise, high self-efficacy, and strong confidence in maintaining long-term exercise behaviors. In terms of rehabilitation needs, they prefer professional instruction, video-based remote guidance, and are motivated by role models. Furthermore, they enjoy comprehensive healthcare accessibility and smooth, multi-channel communication with professionals.

*P4: “It's different from regular exercise; it's planned and goal-oriented, not just random movements. Aerobic exercise, resistance training, and flexibility exercises are all types of exercise rehabilitation.”*

*P9: “I exercise 1-2 hours every morning—playing badminton, kicking shuttlecocks, table tennis... For physical conditioning, professional guidance would be better, with experts giving suggestions, like being guided through warm-ups... The medical conditions here are excellent. We have top tertiary hospitals nearby for major check-ups, and the community clinic staff even come to my home to measure my blood pressure and check on my COPD regularly. It's incredibly convenient.”*

*P18: “I have 100% confidence in maintaining exercise rehabilitation-it has become a habit, just like eating and sleeping. It feels wonderful, especially going to the sports field at night, with the breeze blowing, chatting with old friends...My children often remind me, text or call to check if I've exercised, and sometimes check my step count on WeChat. They all care about me so much.”*

#### 3.3.2 Routine independent exercisers

This subgroup primarily comprises older, urban retirees with disease durations of 8–12 years. Their exercise behaviors were relatively regular but limited in variety, and most activities were performed alone. While they acknowledge the benefits of physical activity, their understanding of exercise rehabilitation remains limited, often equating it with daily walking or simple breathing exercises. They frequently exercise alone, mainly walking in parks or community areas, prefer solitary exercise, and often decline company from family or fellow patients. Despite adequate family support, they lack peer support and have minimal interaction with other patients. Professional support from healthcare providers is also limited, with little guidance on exercise rehabilitation. Psychologically, they generally report feeling calm during exercise activities and exhibit moderate self-efficacy. The core needs of this group focus on safety and convenience. While they do not require real-time supervision, they express a strong desire to learn self-monitoring methods, such as tracking heart rate and blood oxygen levels, and show interest in safety devices like activity trackers. Regarding healthcare resources, their accessibility is limited to basic community care, which is hindered by low trust and poor communication channels with professionals.

*P3: “My daughter bought me this massager. We have an oxygen concentrator, respiratory equipment, nebulizer... My whole family supports my exercise routine and finds ways to facilitate my recovery. I still hope someone can first teach me and monitor my exercise safety, show me what proper form looks like, and what warning signs to watch for. Once I understand the basics, I'll be able to manage about 70-80% of it on my own.”*

*P5: “I walk regularly, almost daily, for about forty to fifty minutes-very consistent. Now I just walk, and occasionally stretch my arms and legs, just moving around casually. As long as the disease doesn't worsen, I feel calm and have no reason to be anxious. I don't have deep conversations with fellow patients; I just greet them and make small talk.”*

*P10: “I only trust the big hospital specialists and have my primary doctor's WeChat for emergencies. As for the community clinic staff? I don't even have their phone numbers, and they don't really know how to guide our rehabilitation exercises anyway.”*

#### 3.3.3 Family-burdened exercisers

This subgroup is predominantly composed of urban or rural residents with relatively low educational attainment, disease durations of 5–8 years, and significant family responsibilities. This subgroup was characterized by monotonous and irregular exercise participation, substantial household and caregiving responsibilities, and a strong preference for social interaction during exercise activities. Their understanding of exercise rehabilitation remains superficial, often equating it with walking or household chores. They typically exercise with neighbors or old friends and rarely engage in activities alone. In terms of social support, these patients face a threefold deficiency: family members offer only verbal encouragement without substantive companionship or participation; peer support is unstable due to competing domestic responsibilities; and they receive minimal specific exercise guidance or follow-up from healthcare professionals. Their rehabilitation needs clearly point toward external support and motivation: they express a strong preference for supervised, safe exercise with professional guidance, ideally delivered in small-group, in-person settings, and rely on family recognition or activity-logging mechanisms as motivational strategies. Regarding healthcare resources, they experience limited accessibility and poor communication channels with medical professionals.

*P1: “Isn't exercise rehabilitation just about being more active, taking walks, and doing housework? I know the benefits; it can reduce flare-ups. Extreme heat or cold bothers me-it makes me feel suffocated and afraid I can't catch my breath.”*

*P6: “I'm somewhat afraid of loneliness, so I prefer group exercises. Usually, we have a whole group of old friends during workouts, and it's so lively! Exercising alone is boring, but together we chat while practicing, time passes quickly, our mood improves, and we don't even feel tired.”*

*P8: “They tell me, ‘Mom, you should go out more,' but then my grandson calls, ‘Grandma, I'm hungry,' or my son asks me to find something—tasks pile up. They're tired from work and can't specifically accompany me. If they could take over my share of housework so I could catch my breath, I could go downstairs for a walk myself, which would be the biggest support. No doctors from the clinic ever contact or check on how I exercise at home. It would be so much better if a professional instructor could lead us in a patient group.”*

#### 3.3.4 Fear-avoidant resource-limited exercisers

This subgroup primarily consists of individuals with low educational levels and disease durations of 1–3 years, who typically reside in rural areas, though may temporarily stay in urban communities for caregiving or family visits. Their defining characteristic is a passive exercise behavior pattern driven by extreme fear of exercise-induced risks and severe resource constraints. Cognitively, they possess a vague understanding of exercise rehabilitation, perceiving its value through a loss-framed perspective, such as “the body will deteriorate without movement” rather than recognizing the proactive benefits of sustained exercise. Their exercise involves passive participation, monotonous routines, and irregular schedules influenced by weather and physical condition. Exercise is confined to familiar, safe areas like village streets and is conducted predominantly alone. These patients face a multidimensional support deficit: absence of family support due to distant children or changed marital status, inability to identify or access peer support networks, and, critically, insufficient professional exercise rehabilitation guidance or follow-up from primary healthcare providers at the township level. Psychologically, they mainly feel fear and loneliness, coupled with low exercise self-efficacy. Their exercise rehabilitation needs to focus on low-intensity, low-balance activities that strictly require in-person supervision and external motivation. Furthermore, they face significant barriers to healthcare access, characterized by blocked communication channels and isolated, passive information acquisition.

*P7: “I usually just walk around the residential area downstairs. Going too far isn't safe, firstly because no one accompanies me, and secondly because I'm afraid of falling. I often worry if I faint outside, who would help me?”*

*P11: “My son and daughter are working away from home, always busy. When they call, it's just to ask about their kids. They've never mentioned exercise rehabilitation. They think that since I can still take the children to and from school, my health must be fine. There are a few old friends in the village who also have breathing problems, but we rarely discuss this. When we meet, we just chat and bask in the sun. Who talks about this stuff? None of us really understands it.”*

*P17: “The village doctors never mention exercise rehabilitation. Going to the city hospital is a massive headache—it's too far, transportation is expensive, and I can't even figure out those touchscreen registration machines without my children.”*

## 4 Discussion

### 4.1 Diversity in exercise rehabilitation behaviors among stable COPD patients

This study identified four distinct types of exercise rehabilitation behavior personas. These four types do not exist in isolation but rather form a continuous spectrum ranging from high adaptability and high resource availability to low adaptability and low resource availability. At one end of the spectrum, “Proactive Self-Managed Exercisers” excel in cognition, exercise behavior, social support, and psychological state, serving as role models in community-based exercise rehabilitation programs. At the opposite end, “Fear-Avoidant Resource-Limited Exercisers” represent the group most in need of targeted nursing interventions and support. The “Routine Independent Exercisers” and the “Family-Burdened Exercisers” types occupy intermediate positions along this spectrum. This suggests that a patient's exercise rehabilitation behavior persona is not fixed but may shift dynamically in response to changes in their health status, psychological state, social support network, and access to medical resources.

### 4.2 Persona characteristics and tailored strategies for exercise rehabilitation behaviors in stable COPD patients

#### 4.2.1 Leveraging proactive patients as peer models to motivate others

For this group, at the micro level, they demonstrate scientific cognition, positive emotions, and high self-efficacy; at the meso level, they benefit from robust family and peer support; at the macro level, they enjoy accessible healthcare resources and smooth communication channels. This multi-level synergy explains their sustained, autonomous engagement in exercise rehabilitation. Consistent with previous studies, higher levels of illness perception (32) and self-efficacy (33) have been identified as key factors in sustaining good exercise habits among patients with chronic conditions. In addition, the findings suggest that higher levels of health literacy may be associated with more favorable exercise rehabilitation behaviors among older patients with stable COPD. Participants in this subgroup demonstrated better disease understanding, stronger exercise confidence, and greater initiative in accessing rehabilitation information and resources. Previous studies have shown that health literacy is closely related to self-management and rehabilitation adherence in chronic disease populations (34). Moreover, strong social support, particularly multi-layered support from family, peers, and healthcare professionals, has been shown to significantly enhance treatment adherence and improve health outcomes (35). Therefore, strengthening patient education and improving access to rehabilitation information may help enhance long-term exercise participation, especially among patients with limited disease knowledge or low exercise confidence. Based on the persona of this patient group, community health programs may consider hosting regular experience-sharing sessions or “Exercise Star” events for COPD patients. Inviting these proactive patients as peer models to share their success stories and positive experiences can reinforce their exercise behaviors through social recognition and motivational incentives (36). Additionally, healthcare providers should develop personalized, progressive exercise prescriptions for them, incorporating not only routine aerobic exercises but also resistance, flexibility, and balance training.

#### 4.2.2 Facilitating exercise diversification while respecting autonomy

For this group, they exhibit highly monotonous exercise patterns yet possess a strong desire to acquire self-monitoring skills for exercise safety. At the meso level, they receive adequate family support but lack peer interaction and professional guidance. At the macro level, their health information sources remain limited. Existing research indicates that long-term engagement in monotonous exercise can lead to boredom and fatigue, potentially hindering comprehensive physical fitness development (37). Moreover, combined exercise regimens have been shown to be more effective than single-form exercises in improving lung function, exercise tolerance, and quality of life in COPD patients (38, 39). Community health initiatives should provide targeted education to introduce the concept of combined exercise, encouraging patients to gradually incorporate resistance or flexibility training 2–3 times weekly alongside walking. Healthcare workers can also train patients to use self-monitoring tools like pulse oximeters and educate them on responding to symptoms, fostering trust over time. Furthermore, given this group's preference for video-based guidance, healthcare providers may establish WeChat groups or utilize digital health platforms to share exercise demonstration videos regularly, enabling patients to voluntarily log activities and receive remote feedback and guidance.

#### 4.2.3 Embedding structured support into fragmented daily routines

For this group, at the micro level, they bear heavy household burdens yet demonstrate a strong desire for social interaction through exercise. At the meso level, they face a threefold support deficit: verbal family support, unstable peer support, and largely absent professional guidance. At the macro level, their access to healthcare resources and information channels remains limited. Recent researches suggest that “exercise snacks”, which are brief exercise sessions spread throughout the day (40), impose low demands on the environment and equipment. This approach may be suitable for those with busy schedules due to its low time commitment and easy integration into daily life (41, 42). Healthcare providers can design “exercise snack plans” integrated into household tasks-such as heel raises while washing dishes, waist twists while wiping tables-to blend movement seamlessly into daily routines. Additionally, community centers should host regular small-group exercise sessions, encouraging patients to invite neighbors or friends and reserving time for social interaction to meet their social needs. Finally, family caregivers should be actively engaged through health education, guiding them to evolve from verbal supporters into active participants by walking together, watching rehabilitation videos, or offering emotional recognition to better motivate this patient subgroup.

#### 4.2.4 Addressing fear-avoidance beliefs and bridging resource gaps

For this group, at the micro level, they are dominated by fear-driven emotions and extremely low exercise self-efficacy, perceiving rehabilitation through a loss-framed perspective. At the meso level, they face a lack of family, peer, and professional support. At the macro level, they contend with severely limited rural healthcare resources, blocked communication channels, and an isolated information environment. Notably, despite their short disease duration, this group shows the worst exercise rehabilitation status. Existing research confirms that a disease duration of < 5 years constitutes a risk factor for poor rehabilitation adherence in COPD patients (43). This may be due to exercise-induced dyspnea and panic, which are new and intense experiences for newly diagnosed patients, fostering a “fear-avoidance” belief that exercise is dangerous (11), particularly for this subgroup whose GOLD Grade 2 impairment early in the illness trajectory causes exertional dyspnea to be perceived as an acute, life-threatening crisis rather than a manageable symptom due to a lack of psychological adaptation.

Addressing the severe challenges of this group, interventions may need to prioritize addressing profound fear while concurrently bridging critical resource gaps. First, improving access to basic rehabilitation resources such as pulse oximeters and exercise equipment in rural primary clinics may help support exercise participation. Second, the widespread lack of targeted exercise guidance and follow-up from primary healthcare providers at the township level must be addressed, consistent with documented low rates of rehabilitation knowledge among primary care professionals in China (44). Additional pulmonary rehabilitation training for primary care providers may improve their ability to support patients. Furthermore, aligning with patients' loss-framed perspective, health education should emphasize the consequences of physical inactivity, like recurrent acute exacerbations and hospitalizations, rather than just the benefits of exercise. Incentives such as medical cost reductions could be offered to patients who achieve exercise goals. Most critically, multidisciplinary teams should be established to address patients' fear-avoidance beliefs through targeted psychological interventions, including Acceptance and Commitment Therapy (45) and motor imagery training (46). Concurrently, as research identifies exercise self-efficacy as a mediating factor in overcoming exercise-related fear (47), healthcare providers can boost patients' confidence by offering positive feedback, tracking exercise progress, and assessing pulmonary improvements. Starting with safe activities like indoor walking and setting highly achievable goals can promote sustained exercise behavior.

### 4.3 Contextual and cultural influences on persona formation and transferability

The transferability of these four personas is bounded by China's specific socio-cultural and healthcare contexts. Culturally, the “Family-Burdened Exercisers” persona reflects traditional Chinese intergenerational dynamics and family-based eldercare expectations (48), where older adults commonly prioritize domestic labor and grandchild-rearing over personal pulmonary rehabilitation. Structurally, the behavioral patterns and resource barriers are deeply tied to China's primary care landscape. Although COPD was included in the National Basic Public Health Service Program in September 2024—tasking community health centers with screening, follow-up, and rehabilitation guidance—this initiative is in its infancy (49). Consequently, in Western systems with mature General Practitioner networks or cultures emphasizing individual autonomy over family interdependence, the characteristics and prevalence of these personas may vary, requiring careful cross-cultural calibration.

### 4.4 Limitations and strengths

This study provides a novel and in-depth understanding of the behavioral heterogeneity in exercise rehabilitation among patients with stable COPD by constructing user personas based on a descriptive qualitative approach. The identified personas may serve as an exploratory framework for understanding variations in rehabilitation behaviors, support contexts, and rehabilitation needs across different patient groups. Nevertheless, several limitations should be acknowledged. First, although data saturation was achieved, the sample size was relatively small (*n* = 18) and recruited from a single community health center in central China. Given the potential influences of distinct cultural values, local customs, and healthcare resources, the generalizability of these findings to other regions or different healthcare settings may be limited. Second, the user personas were initially developed based on qualitative data and have not yet been validated through quantitative methods or external samples. Third, this study only captured the perspectives of patients themselves. Other key stakeholders who also influence patients' exercise behaviors, such as family caregivers and primary care nurses, were not included. Their insights could further enrich the persona profiles and intervention strategies.

### 4.5 Recommendation for future research

Future research should validate and refine these user personas through larger, multi-center studies incorporating quantitative methods such as structured scales or scoring systems to further quantify and verify the classification criteria. Additionally, integrating data-driven approaches, such as using electronic health records or wearable device data, could help optimize the persona models and enhance their predictive accuracy. It is also recommended that future studies include the views of other stakeholders, particularly family caregivers and primary care nurses, to gain a more comprehensive understanding of the facilitators and barriers to exercise rehabilitation in community-dwelling older COPD patients. Moreover, interventional studies are needed to evaluate the effectiveness of tailored rehabilitation strategies based on these user personas, especially their impact on exercise adherence, physiological outcomes, and quality of life. Finally, exploring the application of digital health technologies, including AI-driven mobile health platforms or tele-rehabilitation, to support personalized exercise prescription and remote monitoring for different patient personas.

## 5 Conclusion

This study developed four user personas representing the exercise rehabilitation behavior patterns of community-dwelling older patients with stable COPD. It revealed that their exercise rehabilitation behaviors were influenced by multidimensional interactions among individual perceptions, social support, rehabilitation needs, and healthcare resources, exhibiting substantial heterogeneity. Based on the distinct characteristics of each persona type, targeted intervention recommendations were proposed, aiming to enhance patients' exercise rehabilitation engagement and self-management capabilities.

## Data Availability

The original contributions presented in the study are included in the article/Supplementary material, further inquiries can be directed to the corresponding author.
