# Supplementary material for: User personas for exercise rehabilitation behaviors in older patients with stable chronic obstructive pulmonary disease: a qualitative study
Source: Front Public Health. 2026 Jun 19;14:1847534. doi: 10.3389/fpubh.2026.1847534 (PMC13330973; doi:10.3389/fpubh.2026.1847534)
Supplement: Supplementary file 1 [file Table_1.docx]

**Supplementary File 1 Consolidated criteria for reporting qualitative studies (COREQ) checklist for qualitative studies**

| **No. Item** | **Guide questions/description** | **Reported on Page** |
| --- | --- | --- |
| **Domain 1: Research team and reflexivity** | | |
| *Personal Characteristics* | | |
| 1. Interviewer/facilitator | Which author/s conducted the interview or focus group? | 2.5 Data collection  P4, line 147 |
| 2. Credentials | What were the researcher’s credentials? E.g. PhD, MDa | 2.5 Data collection  P4, line 147 |
| 3. Occupation | What was their occupation at the time of the study? | 2.5 Data collection  P4, line 147 |
| 4. Gender | Was the researcher male or female? | 2.5 Data collection  P4, line 147 |
| 5. Experience and training | What experience or training did the researcher have? | 2.5 Data collection  P4, line 147-149 |
| *Relationship with participants* | | |
| 6. Relationship established | Was a relationship established prior to study commencement? | We didn’t establish a relationship with participants prior to study commencement. |
| 7. Participant knowledge of the interviewer | What did the participants know about the researcher? e.g. personal goals, reasons for doing the research | 2.5 Data collection  P4, line 154-155 |
| 8. Interviewer characteristics | What characteristics were reported about the interviewer/facilitator? e.g. Bias, assumptions, reasons and interests in the research topic | 2.5 Data collection  P4, line 149-151 |
| **Domain 2: study design** | | |
| *Theoretical framework* | | |
| 9. Methodological orientation and Theory | What methodological orientation was stated to underpin the study? e.g. grounded theory, discourse analysis, ethnography, phenomenology, content analysis | Content analysis 2.6.1 Extraction of persona label dimensions  P5, line 173 |
| *Participant selection* | | |
| 10. Sampling | How were participants selected? e.g. purposive, convenience, consecutive, snowball | Purposive  2.2 Study setting and recruitment  P4, line 122 |
| 11. Method of approach | How were participants approached? e.g. face-to-face, telephone, mail, email | Face to face  2.4 Interview guideline  P4, line 137 |
| 12. Sample size | How many participants were in the study? | 2.5 Data collection  P5, line 168 |
| 13. Non-participation | How many people refused to participate or dropped out? Reasons? | 2.5 Data collection  P4, line 155-156 |
| *Setting* | | |
| 14. Setting of data  collection | Where was the data collected? e.g. home, clinic, workplace | Community health centre 2.2 Study Setting and Recruitment  P4, line 121 |
| 15. Presence of non-  participants | Was anyone else present besides the participants and researchers? | 2.5 Data collection  P4, line 159-160 |
| 16. Description of sample | What are the important characteristics of the sample? e.g. demographic data, date | 3.1 Participant Characteristics  P6, line 233-240, Table 1 |
| *Data collection* | | |
| 17. Interview guide | Were questions, prompts, guides provided by the authors? Was it pilot tested? | 2.4 Interview guideline  P4, line 138-140 |
| 18. Repeat interviews | Were repeat interviews carried out? If yes, how many? | No 2.5 Data collection  P5, line 168-169 |
| 19. Audio/visual recording | Did the research use audio or visual recording to collect the data? | 2.5 Data collection  Audio recording  P4, line 153 |
| 20. Field notes | Were field notes made during and/or after the interview or focus group? | Yes 2.8 Rigour and reflexivity  During the interview  P6, line 230 |
| 21. Duration | What was the duration of the inter views or focus group? | Between 20 and 40 minutes 2.5Data collection  P4, line 159 |
| 22. Data saturation | Was data saturation discussed? | 2.5 Data collection  P4-P5, line 162-169 |
| 23. Transcripts returned | Were transcripts returned to participants for comment and/or correction? | 2.6.1 Extraction of persona label dimensions  P5, line 172-173 |
| **Domain 3: analysis and findings** | | |
| *Data analysis* | | |
| 24. Number of data  coders | How many data coders coded the data? | 2.6.1 Extraction of persona label dimensions  P5, Line 173-174 |
| 25. Description of the  coding tree | Did authors provide a description of the coding tree? | 2.6.1 Extraction of persona label dimensions |
| 26. Derivation of themes | Were themes identified in advance or derived from the data? | Derived from the data. |
| 27. Software | What software, if applicable, was used to manage the data? | NVivo 11.0  2.6.1 Extraction of persona label dimensions  P5, line 173 |
| 28. Participant checking | Did participants provide feedback on the findings? | Yes  2.6.1 Extraction of persona label dimensions  P5, line 172-173 |
| *Reporting* | | |
| 29. Quotations presented | Were participant quotations presented to illustrate the themes/findings? Was each quotation identified? e.g. participant number | Yes  3.3.1-3.3.4  P8-P11 Supplementary File 3 |
| 30. Data and findings  consistent | Was there consistency between the data presented and the findings? | Yes,  3. Results |
| 31. Clarity of major  themes | Were major themes clearly presented in the findings? | Yes  3. Results |
| 32. Clarity of minor  themes | Is there a description of diverse cases or discussion of minor themes? | Yes   1. Results 2. Discussion |

Developed from:

Tong A, Sainsbury P, Craig J. Consolidated criteria for reporting qualitative research (COREQ): a 32-item checklist for interviews and focus groups. *International Journal for Quality in Health Care* 2007;19(6):349 – 357
