# Supplementary material for: User personas for exercise rehabilitation behaviors in older patients with stable chronic obstructive pulmonary disease: a qualitative study
Source: Front Public Health. 2026 Jun 19;14:1847534. doi: 10.3389/fpubh.2026.1847534 (PMC13330973; doi:10.3389/fpubh.2026.1847534)
Supplement: Supplementary file 2 [file Table_2.docx]

**Supplementary File 2 Interview Guide - English version**

| **Primary Domain** | **Secondary Topic** | **Prompts & Probes (To be used flexibly to deepen discussion)** |
| --- | --- | --- |
| 1. Microsystem (Individual Level) | 1.1 Understanding & Perceptions | •How did you first learn about exercise rehabilitation?  •In your own words, what do you think exercise rehabilitation involves? How is it different from general physical activity or exercise?  •What potential benefits do you believe it could offer someone with COPD like yourself?  •(If knowledge is limited) Have you ever considered using physical activity to manage your health? What benefits of being active are you aware of? |
|  | 1.2 Current exercise status & Experiences | •What specific activities or exercises do you do? (e.g., walking, Tai Chi, using equipment)  •Where, how often, and for how long?  •What do you usually feel during these activities? (e.g., shortness of breath, fatigue).  •Do you take any preparatory or safety measures? (e.g., warm-up, use of reliever medication) |
|  | 1.3 Psychological status & Emotional Factors | •Psychological: e.g., fear (of breathlessness, falling), low motivation, lack of confidence. |
|  | 1.4 Needs & Preferences (Autonomy & Competence & Relatedness) | •Autonomy: What would your ideal, self-managed exercise rehabilitation look like? (Preferred type, setting, alone/with others, time of day, frequency, duration)  •Competence: What kind of support would help you feel more confident and safe? (e.g., preferred mode of instruction, feedback on progress, safety measures)  •Relatedness: What kind of social or environmental support do you value or desire? (e.g., preference for exercising alone or with others and with whom, sources of encouragement, desired community or hospital-based support services) |
| 1. Mesosystem (Social Network Level) | 2.1 Social Facilitators | What factors have helped your engagement in exercise rehabilitation? •Social Support: e.g., encouragement, reminders, or companionship from family/friends; sharing experiences with peers. •Professional Support: (e.g., advice, monitoring, or follow-up from healthcare professionals)  •Resources: e.g., educational materials, mobile applications, community-based programs or group activities. |
|  | 2.2 Social Barriers | What factors have hindered your engagement in exercise rehabilitation?  •Physical: e.g., breathlessness, fatigue, leg weakness, comorbidities.  •Psychological: e.g., fear.  •Environmental: e.g., limited space, lack of facilities or equipment.  •Knowledge-Related: e.g., uncertainty about appropriate type, intensity, duration, or safety precautions. |
| 1. Macrosystem (Community & Policy Level) | 3.1 Healthcare Resources, Environment & Suggestions | •What healthcare services related to you? (e.g., community health centers, tertiary hospitals)  •What community resources, activities, or environmental factors currently support your exercise?  •What advice would you give to hospitals, community centers, or the government to better support older COPD patients like you? |
|  | 3.2 Communication & Information | •How would you describe your communication with healthcare professionals?  •Where do you usually get information about COPD or exercise? (e.g., healthcare providers, family/friends, TV, short videos, WeChat, brochures) |

**Note:** The design of the interview questions in 1.4: Needs & Preferences is informed by Self-Determination Theory (SDT), a well-established macro-theory of human motivation and personality. Specifically, it draws upon SDT's three Basic Psychological Needs—Autonomy, Competence, and Relatedness—to structure the exploration of older COPD patients' needs and preferences regarding exercise rehabilitation.
