# Supplementary material for: User personas for exercise rehabilitation behaviors in older patients with stable chronic obstructive pulmonary disease: a qualitative study
Source: Front Public Health. 2026 Jun 19;14:1847534. doi: 10.3389/fpubh.2026.1847534 (PMC13330973; doi:10.3389/fpubh.2026.1847534)
Supplement: Supplementary file 3 [file Table_3.docx]

**Supplementary File 3 Patient personas and their patient quotations.**

| **Personas** | **Patient Quotations** |
| --- | --- |
| **Persona 1:** Proactive Self-Managed Exercisers | **P2:** There definitely needs to be a role model to motivate everyone (in exercise rehabilitation). When we patients communicate, we see how well someone is recovering. For example, if someone who couldn't sing before now sings regularly for years with stable condition, I'd think "I should try that too." When I learn someone also has COPD, I'll share my daily experiences with them. So it's human nature, when people see certain results, they tend to imitate and try it themselves.  **P2:** The healthcare conditions are quite good. At least here we have these large tertiary grade-A specialized hospitals—as a frequent patient, I'm often hospitalized, and can always discuss any issues with them. The community services are pretty good too? They provide home visits – checking blood pressure, measuring blood sugar, answering health inquiries – very comprehensive services. For my COPD, the community center even contacts me for annual check-ups.  **P4:** Of course, I've heard of exercise rehabilitation. Don't all hospitals have rehabilitation departments now? It's different from regular exercise... it's planned and goal-oriented, not just random movements. You know, aerobic exercise, resistance training, including flexibility exercises, are all types of exercise rehabilitation.  **P9:** I basically exercise 1-2 hours every morning after waking up – either playing badminton, kicking shuttlecocks, that kind of routine. I like to sweat slightly when doing sports, Sometimes I play table tennis or basketball. I rarely play soccer now, the reason I played soccer was because it was my former profession.  **P9:** For physical conditioning and general fitness, professional guidance would be better, with experts giving suggestions, right? For example, being guided through warm-ups before exercise. Like when I was on the professional soccer team, we always warmed up before training...The medical conditions here are excellent. We have top tertiary hospitals nearby for major check-ups, and the community clinic staff even come to my home to measure my blood pressure and check on my COPD regularly. It's incredibly convenient.  **P15:** My regular routine includes power walking or jogging, using equipment like horizontal bars for pull-ups, and the most consistent practice is Baduanjin-these three exercise methods. I occasionally swim too.  **P15:** Having supervision would help handle acute episodes if they occur. Also, seniors are prone to falls during exercise – having someone watch over us provides peace of mind. It is also very necessary to frequently post some video guidance in the group. Many fellow patients also have comorbidities like diabetes or hypertension—they don't just have one condition. Hypoglycemia in diabetics can be really serious.  **P18:** I have 100% confidence in maintaining exercise rehabilitation, it has become a habit, just like eating and sleeping. When it's time, someone reminds me, and I also want to go out and move. It feels wonderful, especially going to the sports field at night, with the breeze blowing, chatting with my old girlfriends about daily life, all the fatigue from running the business just disappears.  **P18:** My family, my children who are in business, they exercise regularly and often remind me. They frequently text or call to check if I've exercised today. Sometimes they can tell whether I've exercised by checking my step count on WeChat. They all care about me so much and keep emphasizing how beneficial exercise is. |
| **Persona 2:** Routine Independent Exercisers | **P3:** My daughter bought me this massager. We have an oxygen concentrator, respiratory equipment, nebulizer, and even traditional Chinese medicine treatments like moxibustion at home. Yes, we have them all. My daughter has also learned cupping therapy and does it for me. My whole family supports my exercise routine and finds ways to facilitate my recovery.  **P3:** I still hope someone can first teach me and monitor my exercise safety, show me what proper form looks like and what warning signs to watch for. Once I understand the basics, I'll be able to manage about 70-80% of it on my own.  **P5:** I walk regularly, almost daily for about forty to fifty minutes-very consistent. I used to bike, but not anymore now that I'm older, I afraid of falling. Now I just walk, and occasionally stretch my arms and legs, just moving around casually. As long as the disease doesn't worsen, I feel calm and have no reason to be anxious. I don't have deep conversations with fellow patients; I just greet them and make small talk.  **P5:** I don't have deep conversations with fellow patients; I just greet them and make small talk.  **P10:** I feel exercise rehabilitation is more specialized, like targeted training guidance from doctors or experts tailored to my condition. Exercise rehabilitation helps me gradually feel stronger lungs and more energy in my body. I get breathless less often and have fewer difficult moments.  P10: I have my primary doctor's WeChat, and usually contact him when my condition worsens. I don't have contact information for community healthcare staff, and I'm not familiar with them. I only trust the big hospital specialists and have my primary doctor's WeChat for emergencies. As for the community clinic staff? I don't even have their phone numbers, and they don't really know how to guide our rehabilitation exercises anyway "  **P14:** Since I often browse short videos on WeChat, some of which provide disease education, I carefully watch the many COPD health education videos I come across, thinking they help with disease management. So, I believe if we could create videos about our exercise rehabilitation routines, it would have a broader reach, be more engaging, and make people more willing to learn. In our chat groups, when questions from fellow patients get answered, everyone can see the responses and learn something from them. It saves resources too, doesn't it? |
| **Persona 3:**  Family-Burdened Exercisers | **P1:** Isn't exercise rehabilitation just about being more active, taking walks, and doing housework? I know the benefits. It can reduce flare-ups. The doctor also said proper exercise helps with breathing difficulties and makes housework easier. I just haven't been consistent. Extreme heat or cold bothers me, it makes me feel suffocated and afraid I can't catch my breath.  **P6:** I'm somewhat afraid of loneliness, so I prefer group exercises. Usually, we have a whole group of old girlfriends during workouts, it's so lively! Exercising alone is boring, but together we chat while practicing-time passes quickly, our mood improves, and we don't even feel tired.  **P8:** They also tell me, "Mom, you should go out more," "Don't always stay cooped up at home." But the next moment, my grandson calls, "Grandma, I'm hungry," or my son asks me to find something, and all these tasks pile up again. They're also tired from work and rest when they come home, so they can't specifically accompany me to exercise. If they could take over my share of housework so I could catch my breath, I could go downstairs for a walk myself-that would be the biggest support for my exercise.  **P8:** Exercising daily isn't feasible – I don't have the time and I'm always busy at home. Once or twice a week would be manageable, preferably lower intensity activities that aren't too exhausting. I'd be willing to try those that don't leave me breathless or sweaty...No doctors from the clinic ever contact or check on how I exercise at home. It would be so much better if a professional instructor could lead us in a patient group.  **P12:** You mean check-ins, right? I'm familiar with that because I often see my granddaughter completing assignments-they do these check-in chains in group chats posting videos. If it's about motivating me to exercise, I think this check-in method is something I'm familiar with and willing to follow.  **P16:** Having one instructor guide a team would work well too. This way, everyone learns and improves through instruction, and we can discuss which movements are incorrect among ourselves - this kind of exercise rehabilitation guidance approach is quite good. |
| **Persona 4:**  Fear-Avoidant Resource-Limited Exercisers | **P7:** If I don't exercise, my body just stiffens up even more. When I move around, I feel the tightness in my chest eases a bit, and I can breathe slightly deeper. It's also for my diabetes and legs-to prevent them from giving out completely.  **P7:** I usually just walk around the residential area downstairs. Going too far isn't safe, firstly because no one accompanies me, and secondly because I'm afraid of falling. I often worry-if I faint outside, who would help me?  **P11:** If you're asking about me doing exercise rehabilitation on my own? (Shakes head) I'd say my confidence is about three out of ten, at most. There's nothing to look forward to, and I don't know what the right way to exercise is. With no one to guide or check on me, I just let it be.  **P11:** My son and daughter are working away from home, always busy. When they call, it's just to ask about their kids-they've never mentioned exercise rehabilitation. They think since I can still take the children to and from school, my health must be fine. There are a few old friends in the village who also have breathing problems, but we rarely discuss this. When we meet, we just chat and bask in the sun—who talks about this stuff? None of us really understand it.  **P17:** The village doctors never mention exercise rehabilitation. Going to the city hospital is a massive headache—it's too far, transportation is expensive, and I can't even figure out those touchscreen registration machines without my children.  **P13:** The village residential area promised to install exercise equipment but never did. There's insufficient supply of exercise rehabilitation equipment. I've seen urban communities have them, and noticed elderly people using equipment there to stretch their legs-it doesn't need to be complicated, just sturdy and durable. Those wristbands that can monitor heart rate would be even better.  **P17:** Sometimes I glance at the disease awareness posters on the walls at the village clinic. The local doctors don't say much about it, and there's little guidance on exercise. If I want to get checked at a big hospital, that's way too troublesome. It's far, transportation is expensive, and I can't figure out those registration and payment machines by myself. I have to call my son or daughter and make them take time off work specifically to bring me. |
