# Supplementary material for: User personas for exercise rehabilitation behaviors in older patients with stable chronic obstructive pulmonary disease: a qualitative study
Source: Front Public Health. 2026 Jun 19;14:1847534. doi: 10.3389/fpubh.2026.1847534 (PMC13330973; doi:10.3389/fpubh.2026.1847534)
Supplement: Supplementary file 4 [file Table_4.docx]

**Supplementary File 4 Exemplary coding tree for Persona 1**

| **Dimension** | **Label** | **Code** | **Representative Raw Quotation** |
| --- | --- | --- | --- |
| Perceptions of exercise rehabilitation | Cognitive Depth | Systematic understanding of rehabilitation | P4: “Of course, I've heard of exercise rehabilitation. Don't all hospitals have rehabilitation departments now? It's different from regular exercise... it's planned and goal-oriented, not just random movements. You know, aerobic exercise, resistance training, including flexibility exercises, are all types of exercise rehabilitation.” |
|  | Perceived Benefits | Multi-dimensional benefits articulated | P18: “It feels wonderful, Exercise rehabilitation is not only beneficial to my heart and lung functions, but also helps lower my blood lipid and blood sugar levels. There are many benefits for me.” |
| Current exercise status | Exercise type | Diverse; walking, ball sports, swimming, Baduanjin | P15: “My regular routine includes power walking or jogging, using equipment like horizontal bars for pull-ups, and the most consistent practice is Baduanjin—these three exercise methods. I occasionally swim too.”  P9: “I basically exercise... either playing badminton, kicking shuttlecocks, that kind of routine. Sometimes I play table tennis or basketball.” |
|  | Frequency | Highly regular, daily | P9: “I basically exercise 1-2 hours every morning after waking up...” |
|  | Duration | Moderate duration | P15: “...The exercise time is moderate, 1-2 hours every night...” |
|  | Locations | Flexible: home, park, gym | P18: "... I can exercise anywhere. Sometimes at home, or in the nearby park, and sometimes at the gym..." |
|  | Companionship | Often with family/friends/peers | P18: “...chatting with my old friends about daily life, all the fatigue from running the business just disappears.”  P4: “Doing exercises and taking a walk with peers or family members is a pleasant thing.” |
| Psychological status | Exercise emotions | Positive (pleasure, excitement) | P18: "It feels wonderful, especially going to the sports field at night... all the fatigue from running the business just disappears." |
|  | Exercise self-efficacy | High | P18: "I have 100% confidence in maintaining exercise rehabilitation, it has become a habit, just like eating and sleeping." |
|  | Loneliness | None | P18: "...I have many friends who exercise with me. Today I call him along, and tomorrow he calls me. I'm not lonely at all..." |
| Exercise rehabilitation needs | Modality | Moderate intensity, varied, challenging activities | P9: "I like to sweat slightly when doing sports, Sometimes I play table tennis or basketball... either playing badminton, kicking shuttlecocks, that kind of routine." |
|  | Guidance | Professional instruction, video-based remote guidance | P9: "For physical conditioning and general fitness, professional guidance would be better, with experts giving suggestions, right? For example, being guided through warm-ups before exercise."  P15: "Having supervision would help handle acute episodes if they occur. Also, seniors are prone to falls during exercise—having someone watch over us provides peace of mind. It is also very necessary to frequently post some video guidance in the group." |
|  | Motivation | Role models | P2: "There definitely needs to be a role model to motivate everyone (in exercise rehabilitation). When we patients communicate, we see how well someone is recovering... I'd think 'I should try that too.' ... When I learn someone also has COPD, I'll share my daily experiences with them." |
| Social support | Family support | Strong instrumental and emotional support | P18: "My family, my children who are in business, they exercise regularly and often remind me. They frequently text or call to check if I've exercised today. Sometimes they can tell whether I've exercised by checking my step count on WeChat." |
|  | Peer support | Active peer engagement | P2: "When we patients communicate, we see how well someone is recovering... When I learn someone also has COPD, I'll share my daily experiences with them. So it's human nature, when people see certain results, they tend to imitate and try it themselves." |
|  | Professional support | Comprehensive | P2: "...as a frequent patient, I'm often hospitalized, and can always discuss any issues with them. The community services are pretty good too? They provide home visits—checking blood pressure, measuring blood sugar, answering health inquiries..." |
| Accessibility of healthcare resources | Healthcare access | Comprehensive; community + tertiary dual support | P2: "The healthcare conditions are quite good. At least here we have these large tertiary grade-A specialized hospitals... The community services are pretty good too? ... For my COPD, the community center even contacts me for annual check-ups." |
|  | Communication status | Smooth interaction; multiple contact channels | P2: "...as a frequent patient, I'm often hospitalized, and can always discuss any issues with them. ... For my COPD, the community center even contacts me for annual check-ups." |
|  | Information sources | Healthcare providers, peers, short videos, TV | P2: "When we patients communicate... I'll share my daily experiences with them."  P18: "...I often browse Douyin and watch sports programs on TV when I have free time. I follow the videos to exercise, especially some of the exercises she said were specifically for us COPD patients with respiratory diseases." |
